# Treatment of Vascular Lesions of the Tongue with Nd:YAG Laser

**DOI:** 10.1155/2009/795363

**Published:** 2009-12-22

**Authors:** Joana Dias Coelho, Vasco Serrão

**Affiliations:** Department of Dermatology, Hospital dos Capuchos, Alameda Santo António dos Capuchos, 1169-050 Lisbon, Portugal

## Abstract

The treatment of vascular lesions of the tongue is a very challenging procedure since the maintenance of the lingual tissue is of critical importance. Numerous treatment options have been described in literature but the Nd:YAG Laser appears to be one of the safest therapeutic options. We described a successful treatment of vascular lesions of the tongue with an excellent clinical result after only one treatment session with the Nd:YAG laser, with conservation of the lingual tissue and its functionality.

## 1. Case Letter

Vascular lesions of the tongue are uncommon and can be associated with complications such as recurrent bleeding (secondary to minor trauma, tongue biting, or dental hygiene) and enlargement of the lesions with difficulty in chewing, swallowing, or speaking. They usually present as soft, compressible, nonpulsatile bluish mass, frequently isolated but may occur as a part of a systemic syndrome (Rendu-Osler-Weber, Blue rubber bleb nevus, and Maffucci's syndrome) [1].

Many different options for treatment are described in literature: surgery, embolization, steroid therapy, cryosurgery, electrodessication, CO2 laser, and Nd:YAG laser [2–6]. These treatments have different clinical and cosmetic results. It is important to prevent the destruction of tongue tissue that may lead to functional disability [7].

The Nd:YAG laser at 1064 nm has a high penetration depth of up to 5-6 mm, since it is selectively absorbed by hemoglobin and poorly absorbed in water. Comparing to the traditional vascular lasers, it penetrates more deeply into the tissue, since light is less absorbed by the chromophore hemoglobin at the 1064 nm wavelength than at 530 to 600 nm wavelength. In addition, the Nd:YAG laser has a coagulative action as it passes through tissues [7–9].

**Figure 1:**
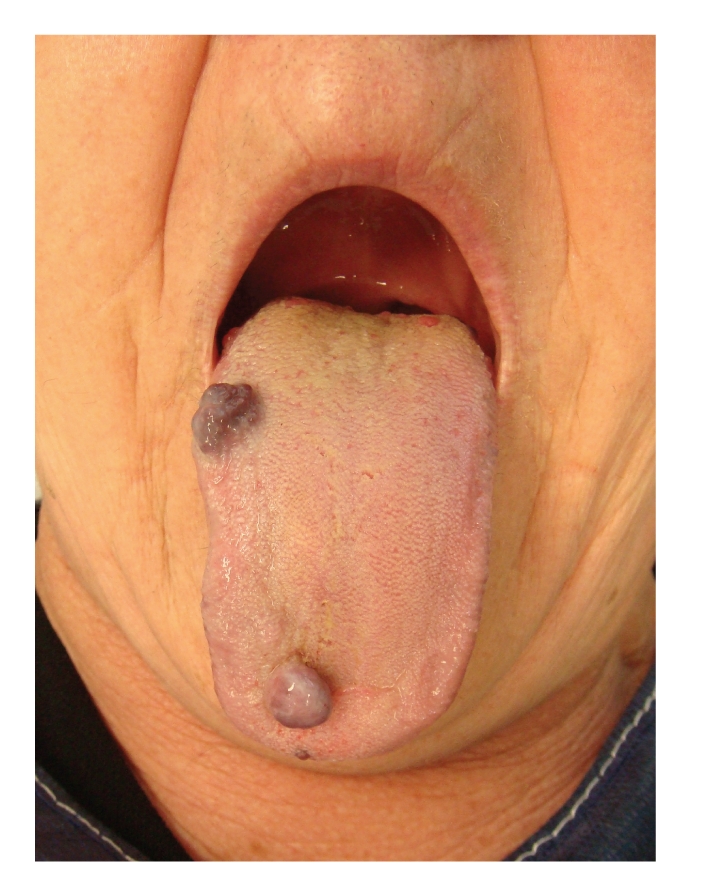
Two soft, dark-red nodules with 12 mm × 10 mm suggestive of vascular lesions and a small nodule of 2 × 2 mm in the tip of the tongue are present.

We describe a case report of a 70-year-old woman with three bluish nodules on her tongue of 5 years' duration (Figure 1). No episodes of recurrent bleeding were present. Physical examination revealed two soft, dark-red nodules with 12 × 10 mm suggestive of vascular lesions and a small nodule of 2 × 2 mm in the tip of the tongue. No histological diagnostic was performed to avoid an unnecessary bleeding.

After proper antiseptic preparation (without local anesthesia), 3 passes of 50 ms with a fluence of 130 J/cm^2^ were used (5 mm spot). Clinical end points were the shrinkage and blanching of the lesion. No adverse reactions were present.

**Figure 2:**
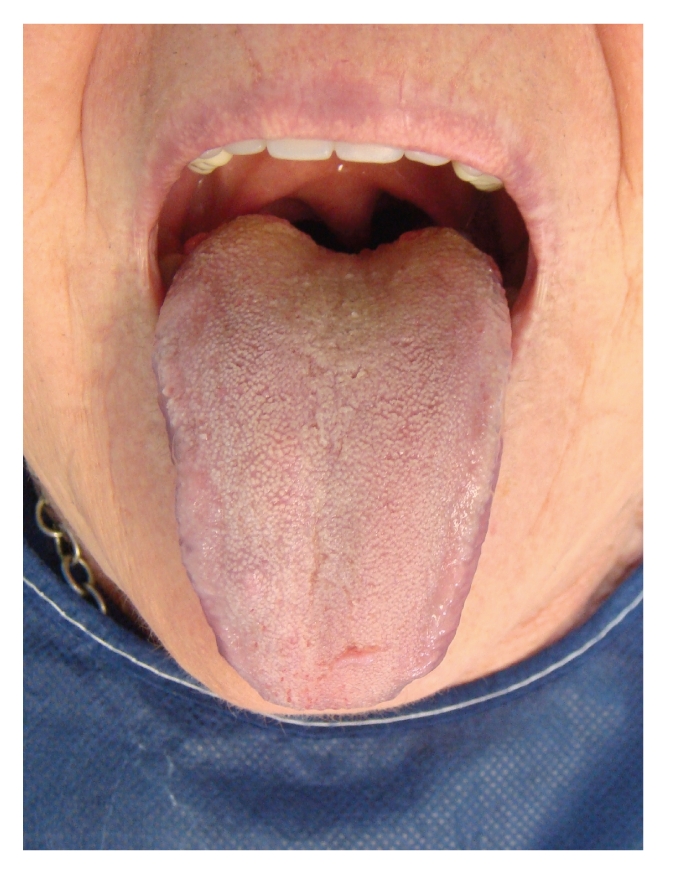
Complete response after only one treatment session.

An excellent result was obtained with only one treatment session. A complete response was present after a 6-month follow-up (Figure 2).

## 2. Discussion

Treatment of vascular malformations of the tongue may be very challenging since the use of a procedure that conserves the lingual tissue and its functionality is essential. 

In literature, there are some reports about the efficacy of the Nd:YAG Laser in the treatment of vascular malformations of the face and neck [10–12]. Classic surgery has a lower risk of recurrence; however, complications, such as bleeding, shortness of breath, or infection and incomplete response can occur [13].

 In our case report, we obtained an excellent result with a complete response to a single treatment. No complications were present. 

We believe that the Nd:YAG Laser is a very good option for the treatment of vascular lesions in the tongue, being a safe, easy, and effective procedure with short downtime and an excellent maintenance of the lingual tissue, especially in thick or nodular lesions.
